# Supplementary material for: A Layered Adopter-Structure Model for the Download of COVID-19 Contact Tracing Apps: A System Dynamics Study for mHealth Penetration
Source: Int J Environ Res Public Health. 2022 Apr 4;19(7):4331. doi: 10.3390/ijerph19074331 (PMC8998972; doi:10.3390/ijerph19074331)
Supplement: Supplementary file 1 [file ijerph-19-04331-s001.zip › ijerph-1632268-supplementary.pdf]

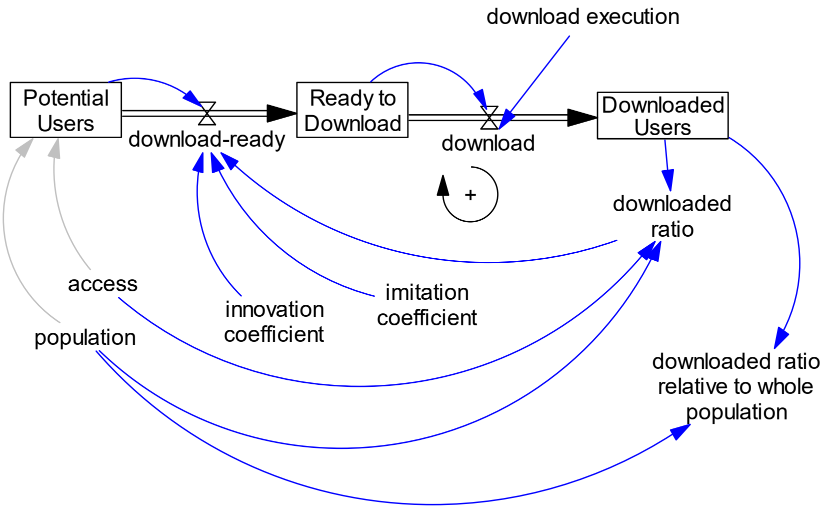

Figure S1. The stock and flow model based on Bass' new product growth model.

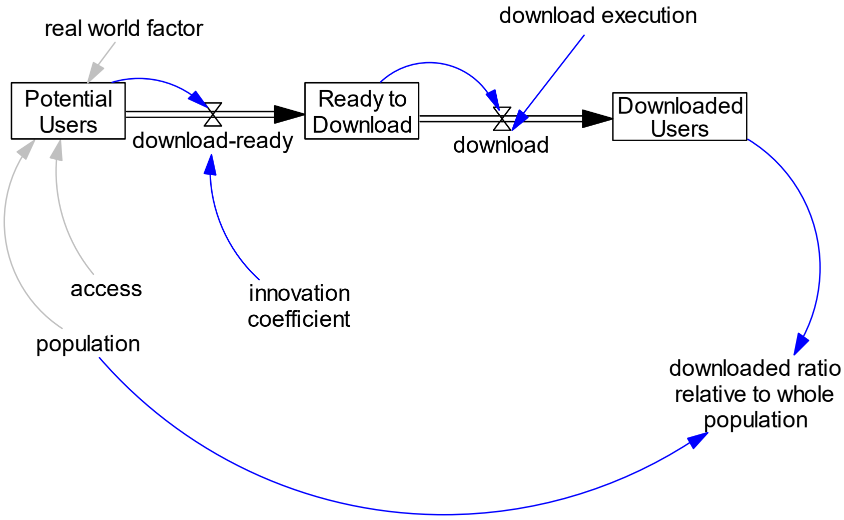

Figure S2. Stock and flow model of the number of downloads of COVIDSafe.

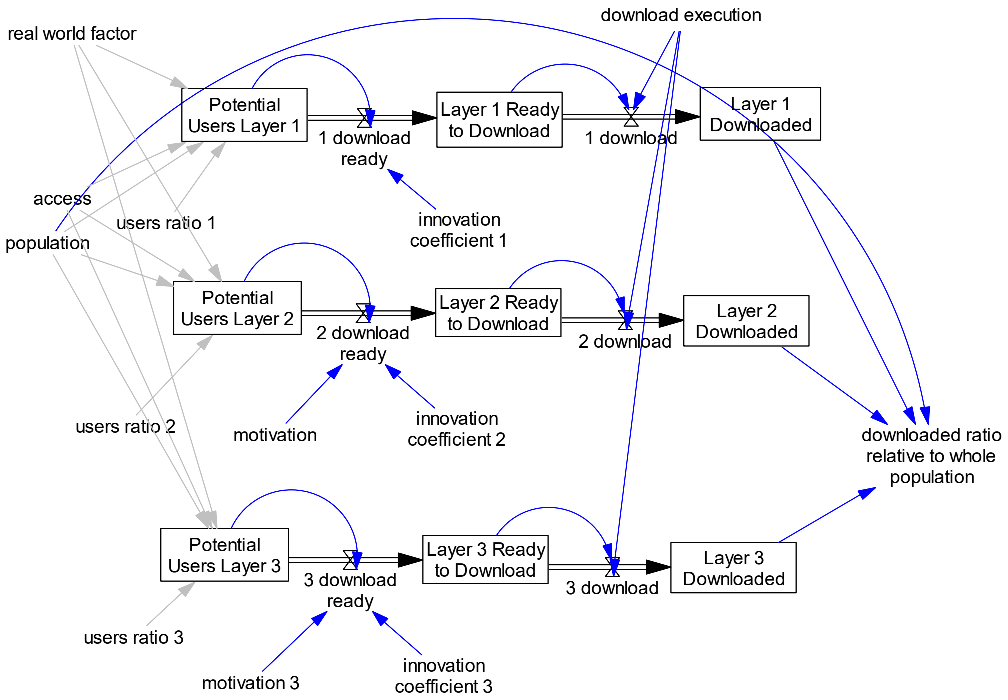

Figure S3. COCOA's stock and flow model for the number of downloads.

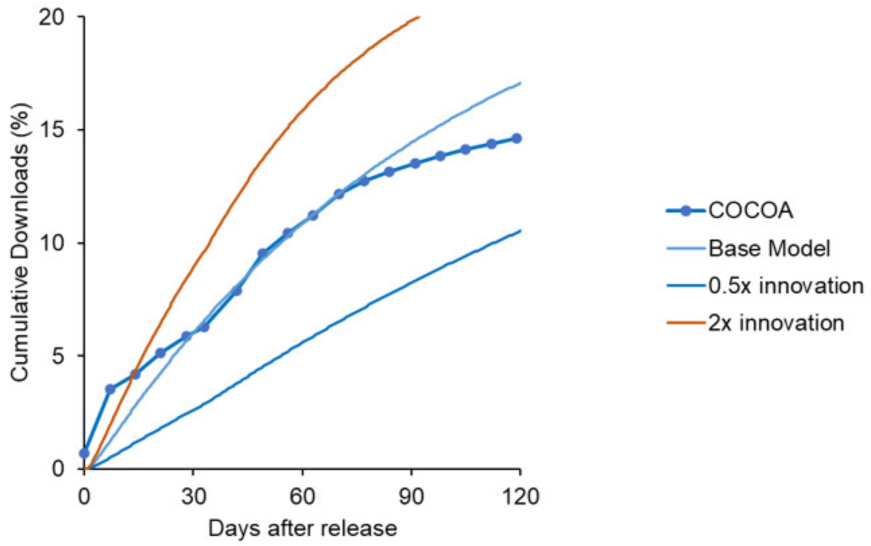

Figure S4. Typical representation of the change in the number of downloads of COCOA described in by single-layered system dynamics model expressed by Figure 4 and Figure S2 with innovation coefficient of 0.01.

Table S1. Parameters of system dynamic model based on Bass' new product growth model.

Stock	Initial Value	Equation
Potential Users	Population * access	-"download-ready"
Ready to Download	0	"download-ready"-download
Downloaded Users	0	Download

Flow	Equation
download-ready	(innovation coefficient + imitation coefficient * downloaded ratio) * Potential Users
download	Ready to Download / download execution

Variable	Value	Source
population	2×10^7	Assumption
access (base model)	0.81	Blom et al.
willing access (willingness model)	0.31 (=0.81 × 0.38)	Blom et al.
innovation coefficient	0.0051	Song et al. (2015)
imitation coefficient	0.4244	Song et al. (2015)
download execution	in 2 days	Assumption
downloaded ratio	Downloaded Users / population	

Table S2. Parameters of COVIDSafe's model of the number of downloads.

Stock	Initial Value	Equation
Potential Users	Population*access*real world factor	-"download-ready"
Ready to Download	0	"download-ready"-download
Downloaded Users	0	Download

Flow	Equation
download-ready	(innovation coefficient + imitation coefficient × downloaded ratio) × Potential Users
download	Ready to Download / download execution

Variable	Value	Source
population	2×10^7	Assumption
access (base model)	0.81 × 0.38	Blom et al.
innovation coefficient	0.2	Calibrated
real world factor	0.8	Assumption
download execution	in 2 days	Assumption
downloaded ratio	Downloaded Users / population	

Table S3. Parameters of COCOA's model for increasing the number of downloads.

Stock	Initial Value	Equation
Potential Users	population*access*real world factor*users ratio	-"download-ready"
Ready to Download	0	"download-ready"-download
Downloaded Users	0	Download

Flow	Equation
download-ready	(innovation coefficient + imitation coefficient * downloaded ratio) * Potential Users
download	Ready to Download / download execution

Variable	Value	Source
population	2×10^7	Assumption
access (base model)	0.81 × 0.38	Blom et al.
innovation coefficient 1	0.06	Calibrated
innovation coefficient 2	0.03	Half of innivation coefficient 1
innovation coefficient 3	0.015	Half of innivation coefficient 2
real world factor	0.8	Assumption
download execution	in 2 days	Assumption
users ratio 1	0.3	Assumption
users ratio 2	0.3	Assumption
users ratio 3	0.3	Assumption
downloaded ratio	Downloaded Users (layer 1, 2, 3) / population	
